# Aspects of mental suffering among nursing professionals at a public hospital in the city of São Paulo, Brazil

**DOI:** 10.47626/1679-4435-2023-1157

**Published:** 2024-11-14

**Authors:** Jessica Andrea Bravo Santana, Rodolfo Antonio Corona, Arthur Arantes Cunha, João Silvestre Silva-Junior

**Affiliations:** 1 Graduate Program in Occupational Medicine, Faculdade de Ciências Médicas da Santa Casa de São Paulo, São Paulo, SP, Brazil; 2 Department of Biological and Health Sciences, Universidade Federal do Amapá, Macapá, AP, Brazil; 3 Department of Medicine, Centro Universitário São Camilo, São Paulo, SP, Brazil

**Keywords:** mental health, mental disorders, occupational health, nurse practitioners, saúde mental, transtornos mentais, saúde do trabalhador, profissionais de enfermagem

## Abstract

**Introduction:**

Nurse practitioners are fundamental to organize patient care at the various levels of health care services. This includes exposure to various work stressors, which can affect the quality of the service as a result of physical and mental exhaustion.

**Objectives:**

This study aimed to investigate and analyze the rate and the factors associated with mental suffering in licensed practical nurses and nursing assistants in a hospital in Brazil.

**Methods:**

This is a cross-sectional study conducted with a nursing support team in a hospital in São Paulo, Brazil. The participants answered the Self-Reporting Questionnaire, which assesses mental suffering, and a questionnaire with questions about sociodemographic, occupational, clinical and health risk behavior data in 2013.

**Results:**

Their mean age was 32.8 years (standard deviation ± 8.5), and their mean length of service was 6.9 years (standard deviation ± 7.0). The participants were predominantly licensed practical nurses (53.5%), women (73.3%), working less than 40 hours per week (63.4%), with more than one job (74.3%), and no chronic illnesses (84.2%). The overall rate of mental suffering was 23.8%. The most frequent (46.5%) complaint was feeling nervous, tense or worried.

**Conclusions:**

Mental suffering is a risk factor for overall health and workers’ health care services should focus on health promotion and disease prevention.

## Abstract (pt)

**Introdução:**

Os profissionais da equipe de enfermagem são parte fundamental da organização do atendimento ao paciente nos diversos níveis de assistência de serviços de saúde. Este trabalho pressupõe a exposição a diversos estressores laborais, que podem comprometer a qualidade do serviço em decorrência do desgaste físico e mental dos trabalhadores.

**Objetivos:**

Este estudo objetivou investigar e analisar a prevalência e os fatores associados ao sofrimento mental entre profissionais técnicos e auxiliares de enfermagem de uma unidade hospitalar brasileira.

**Métodos:**

Trata-se de um estudo transversal realizado com a equipe de suporte de enfermagem em um hospital na cidade de São Paulo, Brasil. Os participantes responderam ao Self-Reporting Questionnaire, que avalia o sofrimento mental, e a um formulário com questões sobre dados sociodemográficos, ocupacionais, clínicos e comportamento de risco para a saúde. Os questionários foram aplicados no ano de 2013.

**Resultados:**

A idade média dos profissionais foi 32,8 anos (desvio-padrão ± 8,5), com tempo médio de exercício da função de 6,9 anos (desvio-padrão ± 7,0). Predominaram participantes técnicos de enfermagem (53,5%), do sexo feminino (73,3%), com jornada de trabalho inferior a 40 horas semanais (63,4%), com mais do que um vínculo empregatício (74,3%) e sem doenças crônicas (84,2%). A prevalência geral de sofrimento mental foi de 23,8%. A queixa mais frequente (46,5%) foi sentir-se nervoso(a), tenso(a) ou preocupado(a).

**Conclusões:**

O sofrimento mental é componente de risco para a saúde geral e deve ser foco de atenção dos serviços de saúde do trabalhador para promoção da saúde e prevenção de doenças.

## INTRODUCTION

In health care, nurse practitioners are the primary link between health services and the community. Nurses, licensed practical nurses, and nursing assistants are key to continuously optimizing the quality of patient care and play an important organizational role at the various levels of care.^1^ In hospitals, they work directly with patients and provide a range of activities, from the simplest, such as hygienic care, comfort, and safety, to preparing patients for consultations, exams, and treatments, to more complex activities, such as planning, organizing, coordinating, and executing direct care and assistance to critically ill, life-threatening patients.^2^

In Brazil, there are approximately two million nurse practitioners; 24.7% are nurses, 57.7% are licensed practical nurses, and 17.6% are nursing assistants, covering all municipalities in all 27 states and all levels of care in the national health system.^3^ The population widely recognizes nurse practitioners for their essential activities for the functioning and maintenance of the quality of health care. However, many of these workers face various work stressors, such as long work shifts, poor working conditions with insufficient or inadequate rest areas, a shortage of materials and equipment, a lack of professional value, and low pay.^1,4,5^

These are factors that lead to occupational stress, favoring the emergence of burnout syndrome, an important psychosocial phenomenon that affects nurse practitioners. Consequently, these workers become physically and/or mentally ill, which can compromise the quality of the service provided to the population.^4,6^

This relationship between poor working conditions and physical and/or mental illness in nurse practitioners can be seen when analyzing the report on the profile of nurse practitioners in Brazil. This report found that 64.2% of nursing assistants and licensed practical nurses and 71.7% of nurses are affected by exhaustion, and a sick leave rate of 19.5% per year.^7^ These workers often face mental suffering, which can be associated with high work-related psychological demand, low control over work, negative self-assessment of their professional performance, and dissatisfaction with career growth.^4-6^

Common mental disorders are defined as mental suffering consisting of symptoms such as anxiety, difficulty concentrating, depression, insomnia, irritability and fatigue, and other somatic symptoms.^4,5^ This condition can compromise the quality of life of workers. Although this condition can compromise the quality of life, functionality, and health, it is not listed in the American Psychiatric Association’s Diagnostic and Statistical Manual of Mental Disorders, 5th edition (DSM-5), or in the International Classification of Diseases and Related Health Problems - 10th Revision (ICD-10).^4,5,8^

Domestic and international studies have increasingly corroborated the link between mental suffering in health care personnel, job characteristics, and the need for sick leave.^4,5^ In Brazil, the social security system officially recognizes hospital care as a major risk factor for mental disorders. In this context, the study of rate and possible factors associated with mental suffering in nurse practitioners is clearly relevant. These workers play a crucial role in any health system, and their sociodemographic, economic, and occupational profile can vary greatly depending on their country or region of residence.^4,5,8^

Thus, considering the social importance of health care teams, the number of workers in Brazil, and the risk of work-related illness, this study aimed to investigate and analyze the rate and factors associated with mental suffering in licensed practical nurses and nursing assistants at a referral hospital in São Paulo, Brazil.

## METHODS

This is a cross-sectional, observational study that analyzed data from nurse practitioners at a referral hospital located in southern area in the city of São Paulo, Brazil in 2013. This is a tertiary care center and primary gateway for urgent and emergency cases in the region, with an average of 15,000 medical visits per month. Although this is a public health unit run by indirect municipal administration, the practitioners are employed as permanent employees pursuant to private sector labor legislation. They work three shifts (morning, afternoon, and night), 6 hours per day, one day off per week for those on the afternoon and morning shifts, and 12-hour shifts with 36 hours of rest for those on the night shift.

The responsibilities of nursing assistants and licensed practical nurses include working in emergency settings, following institutional protocols and guidelines; working as part of the nursing and multidisciplinary team to directly assist patients in low and medium complexity procedures and, when necessary, in high complexity procedures, supervised by a nurse; providing care to patients according to medical and nursing routines, protocols, prescriptions, and guidelines; assisting nurses in planning, programming, guiding, and supervising nursing activities; recording the care provided to patients and their families in specific documents/medical records; complying with the policies, protocols, routines, and procedures of their job and workplace. These tasks are performed in different settings, including drug administration in emergency medical services, operating rooms, wards, clinical analysis laboratories, observation wards, acute care, diagnostic imaging centers, etc.

Of the 119 licensed practical nurses working at the hospital, seven were on sick leave, five were on vacation, five did not return our calls, and only one refused to participate in the study. A total of 101 (84.9%) licensed practical nurses or nursing assistants agreed to participate and answered a set of questions related to sociodemographic characteristics (sex, age, education, marital status, and children), health risk behaviors (smoking and alcohol use), occupational characteristics (job position, work shift, overtime, length of service, length of service at the facility, other jobs, and weekly workload), and clinical characteristics (weight and height to calculate body mass index [BMI], chronic diseases, previous psychiatric treatment, and sleep time).

All 101 practitioners (54 licensed practical nurses and 47 nursing assistants) answered in 2013 the Brazilian Portuguese version of the Self-Reporting Questionnaire (SRQ-20), which is the World Health Organization’s (WHO) self-administered questionnaire for assessing mental suffering.^9^ The SRQ-20 is a multidimensional questionnaire often used in epidemiological studies to screen for mental disorders. The questionnaire consists of 20 dichotomous questions (yes/no questions) which can be grouped into four symptom groups for descriptive evaluation: somatic symptoms, depressive/anxious mood, decreased vital energy, and depressive thoughts.^9,10^ Each positive response scores 1 and the sum of these values determines the final assessment score. In this study, the cut-off point used to determine mental suffering was seven or more positive responses, regardless of sex.^9^

Continuous variables were presented using descriptive statistics, mean and standard deviation (SD). Categorical variables were expressed as absolute and relative frequencies and statistically analyzed using the Statistical Package for Social Sciences version 20.0. The distribution of participants was analyzed according to the dependent variable (mental suffering) and independent variables (sociodemographic, occupational and clinical health risk behaviors), using the chi-square test or Fisher’s exact test. The significance level adopted was p ≤ 0.05.

The project was registered (Certificado de Apresentação de Apreciação Ética No. 10866212.6.0000.5479) on the Plataforma Brasil of the Ministério da Saúde (Brazil Ministry of Health) and the Human Research Ethics Committee of the Irmandade da Santa Casa de Misericórdia de São Paulo approved it (Opinion No. 191.653.13). All participants read and signed the informed consent form.

## RESULTS

The rate of mental suffering in the 101 nursing assistants and licensed practical nurses who participated in the study was 23.8%. The mean age was 32.8 years (SD ± 8.5), from 19 to 53 years. The mean length of service was 6.9 years (SD ± 7.0) and the mean length of service at the hospital was 2 years (SD ± 1.6). Most were women (73.3%), with high school education (67.3%), living with a partner (50.5%), and with one child (68%). Smoking was reported by 10.9% and alcohol use by 3% (Table 1).

**Table 1:** Mental suffering (yes/no) according to sociodemographic and behavioral health risk characteristics: distribution of nurse practitioners at a public hospital in the city of São Paulo, Brazil, 2013 (n = 101)

Variables (n)	n	%	Mental suffering				p-value
			Yes		No		
			n	%	n	%	
Overall participants	101	100.0	24	23.8	77	76.2	
Sex (101)							
Male	27	26.7	3	11.1	24	88.9	0.071^*^
Female	74	73.3	21	28.4	53	71.6	
Age group (101) (years)							
≤ 24	16	15.8	2	12.5	14	87.5	0.479†
25 to 34	53	52.5	16	30.2	37	69.8	
35 to 44	19	18.8	4	21.1	15	78.9	
≥ 45	13	12.9	2	15.3	11	84.7	
Education (101)							
High school	68	67.3	16	23.5	52	76.5	0.937^*^
College degree	33	32.7	8	24.2	25	75.8	
Marital status (101)							
With a partner	51	50.5	14	27.5	37	72.5	0.379^*^
With no partner	50	49.5	10	20.0	40	80.0	
Children (100)							
Yes	68	68.0	19	27.9	49	72.1	0.086^*^
No	32	32.0	4	12.5	28	87.5	
Smoker (101)							
Yes	11	10.9	3	27.3	8	72.7	0.720†
No	90	89.1	21	23.3	69	76.7	
Alcohol use (101)							
Yes	3	2.9	1	33.3	2	66.7	0.561†
No	98	97.1	23	23.5	75	76.5	

* Pearson’s chi-square test.

† Fisher’s exact test.

As for occupational factors, most participants were licensed practical nurses (53.5%) who worked the night shift (45.5%), less than 40 hours a week (63.4%), overtime shifts (53.5%), and had more than one job (74.3%) (Table 2).

**Table 2:** Mental suffering (yes/no) according to occupational characteristics: distribution of nurse practitioners at a public hospital in the city of São Paulo, Brazil, 2013 (n = 101)

Variables (n)	n	%	Mental suffering				p-value
			Yes		No		
			n	%	n	%	
Job position (101)							
Nursing assistant	47	46.5	12	25.5	35	74.5	0.697^*^
Licensed practical nurse	54	53.5	12	22.2	42	77.8	
Work shift (101)							
Morning	25	24.8	5	20.0	20	80.0	0.846^*^
Afternoon	30	29.7	8	26.7	22	73.3	
Night	46	45.5	11	23.9	35	76.1	
Weekly workload (93) (hours)							
< 40	59	63.4	16	27.1	43	72.9	0.083^*^
≥ 40	34	36.6	4	11.8	30	88.2	
Overtime (99)							
Yes	53	53.5	12	22.6	41	77.4	0.690^*^
No	46	46.5	12	26.1	34	73.9	
Other jobs (101)							
Yes	75	74.3	20	26.7	55	73.3	0.244^*^
No	26	25.7	4	15.4	22	84.6	

* Pearson’s chi-square test.

As for clinical characteristics, most participants had a regular weight (60.8%), reported no chronic diseases (84.2%), had not been treated for psychiatric conditions previously (96%), and slept less than 8 hours a day (59.6%) (Table 3).

**Table 3:** Mental suffering (yes/no) according to previous clinical comorbidities and daily sleep time: distribution of nurse practitioners at a public hospital in the city of São Paulo, Brazil, 2013 (n = 101)

Variables (n)	n	%	Mental suffering				p-value
			Yes		No		
			n	%	n	%	
BMI (97)							
Underweight/eutrophic	59	60.8	12	20.3	47	79.7	0.557^*^
Overweight	23	23.7	7	30.4	16	69.6	
Obesity	15	15.5	4	26.7	11	73.3	
Chronic disease (101)							
Yes	16	15.8	5	31.3	11	68.8	0.523^*^
No	85	84.2	19	22.4	66	77.6	
Previous psychiatric treatment (101)							
Yes	4	4.0	1	25.0	3	75.0	0.999^*^
No	97	96.0	23	23.7	74	76.3	
Sleep time/day (94)							
< 8	56	59.6	13	23.2	43	76.8	0.805†
≥ 8	38	40.4	8	21.1	30	78.9	

BMI = body mass index.

* Fisher’s exact test.

† Pearson’s chi-squared test.

This study found no statistically significant associations between the independent variables and the dependent variable (mental suffering) (Tables 1, 2, and 3). When analyzing the answers to the SRQ-20 by symptom group, the following symptoms are worth noting: 36.6% complained of poor sleep (Q3) as one of the somatic symptoms; 46.5% of the participants reported feeling nervous, tense or worried (Q6) as one of the depressive/anxious mood symptoms; 30.7% reported feeling tired all the time (Q18) as one of the symptoms related to lower vital energy; and 13.9% reported having lost interest in things (Q15) as a depressive thought symptom. In the latter group, 3% of respondents reported suicidal ideation (Q17) (Table 4).

**Table 4:** Groups of symptoms and items of the Self-Reporting Questionnaire (SRQ-20): distribution of positive responses from nurse practitioners at a public hospital in the city of São Paulo, Brazil, 2013 (n = 101)

SRQ-20	n	%
Somatic symptoms		
Q1 - Do you often have headaches?	25	24.8
Q2 - Is your appetite poor?	10	9.9
Q3 - Do you sleep badly?	37	36.6
Q5 - Do your hands shake?	16	15.8
Q7 - Is your digestion poor?	21	20.8
Q19 - Do you have uncomfortable feelings in your stomach?	30	29.7
Depressed/anxious mood		
Q4 - Are you easily frightened?	19	18.8
Q6 - Do you feel nervous, tense or worried?	47	46.5
Q9 - Do you feel unhappy?	29	28.7
Q10 - Do you cry more than usual?	10	9.9
Lower vital energy		
Q8 - Do you have trouble thinking clearly?	15	14.9
Q11 - Do you find it difficult to enjoy your daily activities?	24	23.8
Q12 - Do you find it difficult to make decisions?	14	13.9
Q13 - Is your daily work suffering?	9	8.9
Q18 - Do you feel tired all the time?	31	30.7
Q20 - Are you easily tired?	26	25.7
Depressive thoughts		
Q14 - Are you unable to play a useful part in life?	3	3.0
Q15 - Have you lost interest in things?	14	13.9
Q16 - Do you feel that you are a worthless person?	3	3.0
Q17 - Has the thought of ending your life been on your mind?	3	3.0

## DISCUSSION

This study found a lower rate of mental suffering than other studies with hospital nurse practitioners, between 30% and 33.7%.^5,11,12^ However, the results of this study are higher than the 14.9% observed by Carvalho et al.^13^ in nurse practitioners working in primary health care.^13^ This can be partly explained because hospital settings are more likely of a higher burden of stress; nurse practitioners work in a care-oriented way in a context with medium to high-complexity patients at risk of death.^11,14-16^

Although no statistically significant association was found between mental suffering and the variables sex, marital status, and children, it is worth noting that the higher rate identified in this study was in women, those with a partner, and those with children, and may be linked to having a double workload. This condition results in the need to devise strategies to balance time between work and family duties, which can predispose to greater psycho-emotional stress.^4,17^ As for women, the lack of recognition and devaluation may also influence the higher rate of mental suffering in this group.^18^ In addition, women find it easier to identify and communicate/report the symptoms of mental disorders, predisposing them to a higher frequency of diagnosis than men.^19^

Alcântara & Assunção^20^ found higher rates of mental suffering in health care personnel with a history of smoking and alcohol use. In terms of these risk behaviors, work-related stressors and mental disorders, especially depression, can be assumed to influence the use and abuse of alcohol and tobacco.^21^ However, this study found a higher rate of mental suffering in health care personnel who had a history of smoking and alcohol use. The present study also reported a higher rate of mental suffering in users of alcohol and tobacco, with no statistical significance.

Rodrigues et al.^5^ found a higher rate of mental suffering in licensed practical nurses in Bahia, Brazil, compared to nursing assistants. This study, however, found no statistically significant difference. One hypothesis may be that the characteristics and psychosocial burdens are similar between groups in terms of work setting and tasks.^11,22^

As for workload, a higher rate of mental suffering was found in workers working less than 40 hours per week. Although this result was not statistically significant, it is a descriptively different result from that found in most studies. Most studies show higher workloads as a factor associated with mental suffering,^23,24^ which was not found in this study, possibly due to factors inherent to the sample. In addition, one possible rationale for this finding is that workers with poor health conditions may have lower workloads due to being less resilient to work demands.^23^

Silva et al.^25^ found a higher rate of mental suffering in overweight and obese health care personnel. The association between these conditions may be mediated by psychological issues linked to negative self-perception of body image and stigmas.^25,26^ In addition, some pathophysiological conditions related to mental disorders, such as alterations in immune, inflammatory, hormonal pathways, and a lack of energy and physical inactivity, are related to obesity and physical inactivity.^27,28^ Despite this relationship in the literature, the data in our study did not indicate a statistically significant association between overweight/obesity and mental suffering, although a higher rate was found in this group.

Sleep time is important for preserving mental health. Poor sleep and wakefulness patterns due to work-related demands have been identified as a contributing factor to work-related mental disorders.^4^ In this study, a sleep pattern of less than 8 hours was not associated with a higher rate of mental suffering. Workers may be adapted to this condition and are not fatigued as often as expected.

Frequently reporting feeling nervous, tense or worried is consistent with other studies with health care personnel using the SRQ-20,^29^ which may be a common psycho-emotional response in these professionals. Some of them, especially licensed practical nurses and nursing assistants, work under conditions of high psychosocial demand associated with responsibility for patient care and safety, aiming to provide a feeling of well-being.^11,22^ Some aspects and results of their work are subject to requirements that exceed their control and depend on circumstances of the health unit and the process of hierarchization of nurse practitioners. This can lead to a toxic workplace and produce symptoms of anxiety.^11,30^

This study has some limitations. The sample is relevant to the context of the hospital studied, but it was not sufficiently large to observe the expected results at the level of statistical significance required. The internal validity of this study has some strengths, particularly the minimization of bias in measuring the outcome of mental suffering, since an SRQ-20 validated and adapted to the Brazilian context was used and applied individually by trained investigators.

## CONCLUSIONS

The nurse practitioners who participated in this study had a lower rate of mental suffering than those found in other studies with workers who had similar jobs in hospitals. However, this study found an important rate of mental suffering, given that it is a component that affects overall health, well-being, quality of life, and work productivity.

Although no factors were statistically associated with mental suffering, this study found individual characteristics that may represent a risk to the overall health of workers. We found significant rates of alcohol use, smoking, and obesity, and also a high rate of mental suffering and evidence of suicidal ideation in three participants.

Thus, considering the high rate of complaints related to mental suffering in hospital nurse practitioners, such as hopelessness, a sense of guilt, irritability, somatic symptoms, and others, occupational health services are recommended to improve measures and policies to foster the biopsychosocial health of workers.
